# Pneumatosis cystoides intestinalis in a trauma patient presenting with pneumoperitoneum: a case report

**DOI:** 10.1186/s13256-021-03183-9

**Published:** 2021-12-17

**Authors:** Elliott Lebby, Medhat Hanna, Thanh-Lan Bui, Adam Rudd, Whayoung Lee, Roozbeh Houshyar

**Affiliations:** 1 Department of Radiological Sciences, University of California Irvine, 101 The City Dr S Building 55, Suite 201, Orange, CA 92868 USA; 2 Department of Pathology and Laboratory Medicine, Medical Sciences I, D‐440, University of California Irvine School of Medicine, Irvine, CA 92697‐4800 USA

**Keywords:** Pneumatosis cystoides intestinalis, Pneumoperitoneum, Abdominal imaging, Case report

## Abstract

**Background:**

Pneumatosis cystoides intestinalis is a rare and usually benign condition in which multiple thin-walled cysts develop in the submucosa or subserosa of the gastrointestinal tract. While usually asymptomatic, severe cases can result in pneumoperitoneum, which can be managed surgically or medically depending on circumstances. We present a case of a patient with pneumatosis cystoides intestinalis, which presented as pneumoperitoneum following trauma. To our knowledge, there are no other published cases in which a trauma patient with pneumoperitoneum was found to have radiologic evidence of pneumatosis cystoides intestinalis.

**Case presentation:**

We present the case of a 37-year-old Hispanic male admitted to the hospital after being involved in a motorcycle accident. Computed tomography imaging of the abdomen and pelvis with oral and intravenous contrast demonstrated trace pneumoperitoneum, possibly originating from the splenic flexure of the colon without evidence of extravasation of oral contrast. Laparoscopy with conversion to exploratory laparotomy revealed bowel abnormalities at the distal transverse colon and splenic flexure, which were identified as pneumatosis cystoides intestinalis by pathology. There was no evidence of bowel perforation. A panel of abdominal radiologists attended the computed tomography interpretation to note that incidental atraumatic or traumatic rupture of the cysts could have caused the pneumoperitoneum. The patient had an uncomplicated postoperative course and was transferred to another facility per insurance request.

**Conclusions:**

When presenting in the context of trauma, pneumatosis cystoides intestinalis can lead to difficult management decisions. To our knowledge, there are no existing evidence-based guidelines for the scenario of concurrent pneumatosis cystoides intestinalis, blunt abdominal trauma, and pneumoperitoneum in a patient with a benign abdominal exam. This patient’s pneumoperitoneum was likely caused by rupture of preexisting cysts rather than frank bowel perforation. Patients who are asymptomatic, lack signs of clinically worrisome disease, and have a low pretest probability will likely not benefit from surgery and can be medically managed. Thorough discussion between surgeons and radiologists can be helpful when evaluating the clinical significance of a patient’s pneumatosis cystoides intestinalis and aid in the decision to perform surgery.

## Background

The term pneumatosis intestinalis is used to describe the presence of gas in the wall of the small or large intestine. It has many causes ranging from benign to life threatening and may be either primary or secondary to another disease process. One of the forms of pneumatosis intestinalis is pneumatosis cystoides intestinalis (PCI), a rare and benign idiopathic condition in which multiple thin-walled cysts develop in the submucosa or subserosa of the bowel [1]. It is usually asymptomatic and often only incidentally found on radiography or endoscopy [2–4]. When symptomatic, patients can present with abdominal pain, obstruction, and bleeding. In some cases, the cysts can rupture leading to pneumoperitoneum [1]. The resulting pneumoperitoneum can be life threatening and requires surgical correction in severe cases. In mild cases, medical management is appropriate [3, 5].

Given the various manifestations of this condition, the clinical significance can often be misinterpreted, resulting in inappropriate management [6]. Contrast-enhanced abdominal computed tomography (CT) is used to establish the diagnosis and diagnose possible associated complications. We present the case of a patient who was found to have radiologic evidence of PCI with pneumoperitoneum in the setting of trauma. To our knowledge, there are no other published cases on this matter.

## Case presentation

A 37-year-old Hispanic male was hospitalized after being involved in a motorcycle crash. The patient suffered blunt chest and abdominal trauma as well as concussion without loss of consciousness. Upon arrival, the patient had a Glasgow Coma scale score of 15 and was hemodynamically stable. His abdomen was soft, nondistended, and nontender to palpation with no guarding. Labs were significant for hemoglobin count of 14.2 g/dL (normal 13.5–17.5 g/dL), white blood count of 11.8 1000/μL (normal 4.5–11 1000/μL), and lactate of 1.7 mmol/L (normal 0.7–2.1 mmol/L), which remained stable throughout his hospital course.

Initial intravenous contrast-enhanced CT scan of the abdomen/pelvis was performed 10 minutes after patient presentation. The CT scan was interpreted by a radiology resident 15 minutes later and final interpretation by a board-certified emergency radiologist was completed in an additional 1 hour and 25 minutes. The initial CT interpretation indicated nonspecific wall thickening of the proximal jejunum, and occult small bowel injury could not be excluded. Given these findings, a repeat CT of the abdomen/pelvis with intravenous and oral contrast was ordered by the trauma service as per our institution’s standard protocol for evaluation of possible occult bowel injury in blunt abdominal trauma.

**Fig. 1:**
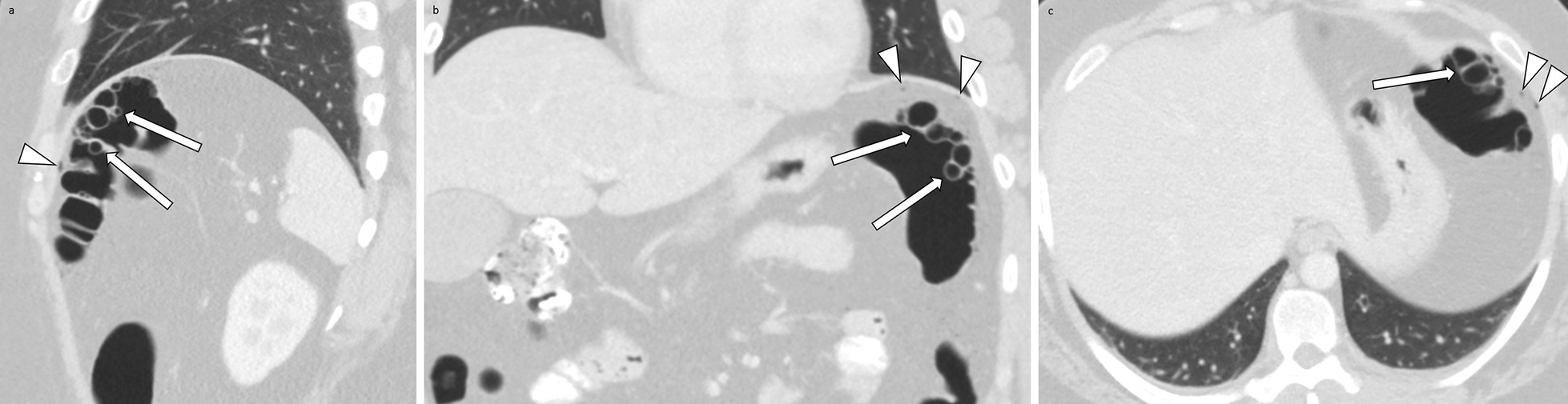
Computed tomography scan of the abdomen/pelvis sagittal view (**a**), coronal view (**b**), and axial view (**c**) demonstrating findings of numerous cysts within the wall of the splenic flexure of the colon (arrows) with trace pneumoperitoneum in the subphrenic spaces (arrowheads)

The follow up CT performed after midnight, 8 hours after the initial CT, demonstrated trace pneumoperitoneum, possibly originating from the splenic flexure of the colon without evidence of extravasation of oral contrast (Fig. 1). The time from repeat CT scan completion to initial interpretation by a radiology resident was 30 minutes. The resident made the surgery team aware of these findings immediately. A board-certified emergency radiologist reviewed and provided another preliminary interpretation for the study 10 minutes later, confirming the presence of pneumoperitoneum. Based on the presence of pneumoperitoneum in a patient with blunt abdominal trauma, the patient was taken to the operating room as per standard protocol at our institution. Six hours after the follow up CT was performed, an academic board-certified abdominal radiologist provided a final interpretation.

**Fig. 2:**
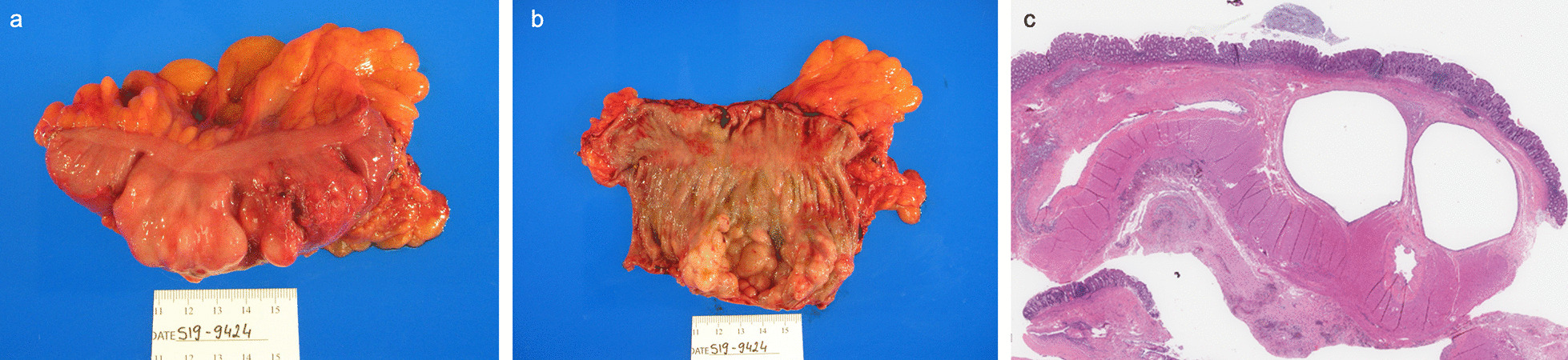
Colonic segment with attached pericolonic fat measuring 12 cm in length and 6 cm in diameter. The serosa is tan–pink and smooth. Upon opening, the mucosa is tan–pink, soft with normal bowel folds. Attached to the folds, multiple large nodules are identified ranging from 0.5 to 3.0 cm. The entire nodular area measures 8.4 × 3.5 × 2 cm. Upon sectioning, the nodules deflate and are consistent with air bubbles (**a**, **b**). Microscopically, the section of the colon showed multiple air-filled cystic structures located in the submucosa. The cysts were lined by macrophages and multinucleated giant cells with bland nuclei. No atypia, pleomorphism, or mitoses were identified. The overlying colonic mucosa exhibited mild reactive changes but did not show significant inflammation or adenomatous change (**c**)

On laparoscopy, cyst-like structures projecting from the wall of the distal transverse colon and splenic flexure were noted. Due to the patient’s body habitus, the surgery team did not feel that they could adequately explore the area and the decision was made to convert to exploratory laparotomy. No evidence of bowel perforation was found. A segmental section of the diseased portion of the colon was taken and sent to pathology. Pathology results showed multiple enlarged cystic nodules ranging from 0.5 to 3 cm in the bowel wall (Fig. 2).

Subsequently, the radiologic studies were reviewed by a panel of abdominal radiologists. The panel concluded that the proximity of the pneumoperitoneum to the pneumatosis cystoides intestinalis suggested that the pneumoperitoneum may have been caused by incidental atraumatic or traumatic rupture of the cysts. However, they could not rule out bowel perforation based on imaging alone.

The patient had an uncomplicated postoperative course and was transferred to an acute care facility of equal acuity for further medical management as per the patient’s health insurance request.

## Discussion

We present the case of a patient who was incidentally found to have pneumatosis cystoides intestinalis in the setting of trauma. Despite relatively benign examination and laboratory, findings, the patient was treated surgically due to concern for bowel perforation.

Due to nonspecific physical examination and laboratory findings, the diagnosis of pneumatosis cystoides intestinalis is generally established through abdominal imaging. Contrast-enhanced CT scan, the test of choice, reveals microvesicular gas collections (10–100 mm cysts or bubbles) within the lamina propria of the bowel. This feature differentiates it from acquired pneumatosis intestinalis, in which linear or curvilinear gas collections can be seen parallel to the bowel wall [4, 7].

Given the various manifestations of this condition, the clinical significance can often be misinterpreted, resulting in inappropriate management [6]. While most cases of PCI can be managed medically, bowel perforation frequently requires urgent surgical intervention [5]. This case is uniquely challenging as the pneumoperitoneum was discovered in the setting of trauma. To our knowledge, there are no existing evidence-based guidelines for the scenario of concurrent PCI, blunt abdominal trauma, and pneumoperitoneum in a patient with a benign abdominal exam.

Although this patient did not display signs of peritonitis on abdominal examination, nor metabolic acidosis, lactate acidosis, or portal venous gas on CT, he was suspected to have a bowel perforation and taken to the operating room for an exploratory laparotomy. Having a high clinical suspicion for bowel perforation in this clinical scenario was rational. However, the overall clinical picture should have been taken into consideration to avoid the error of diagnostic anchoring. In this case, the asymptomatic patient and minimal amount of pneumoperitoneum was most likely caused by rupture of some preexisting PCI cysts due to trauma. When PCI is complicated by pneumoperitoneum, clinicians face the difficult decision of discriminating patients with a relatively benign PCI from patients with life-threatening bowel perforation.

## Conclusion

As illustrated by this case, it is important for clinicians to recognize the entity of pneumatosis cystoides intestinalis and be able to differentiate benign from clinically worrisome disease. Asymptomatic patients who lack signs of clinically worrisome disease and who have a low pretest probability will likely not benefit from surgery and can be medically managed. Surgeons and radiologists should have a thorough discussion to evaluate the clinical significance of a patient’s PCI and weigh the benefits and risks of surgery.

## Data Availability

Not applicable.

## Abbreviations

PCI: Pneumatosis cystoides intestinalis
CT: Computed tomography
